# Identification of a Biostimulating Potential of an Organic Biomaterial Based on the Botanical Extract from *Arctium lappa* L. Roots

**DOI:** 10.3390/ma14174920

**Published:** 2021-08-29

**Authors:** Agnieszka Szparaga, Sławomir Kocira, Ireneusz Kapusta

**Affiliations:** 1 Department of Agrobiotechnology, Koszalin University of Technology, Racławicka 15-17, 75-620 Koszalin, Poland; agnieszka.szparaga@tu.koszalin.pl; 2 Department of Machinery Exploitation and Management of Production Processes, University of Life Sciences in Lublin, Akademicka 13, 20-950 Lublin, Poland; 3 Department of Food Technology and Human Nutrition, College of Natural Science, University of Rzeszow, Zelwerowicza 4., 35-601 Rzeszow, Poland; ikapusta@ur.edu.pl

**Keywords:** biogranulate, biostimulant, biomaterial, soybean, burdock

## Abstract

The development of novel biomaterials based on plant extracts is expected to boost yields without adversely affecting environmental diversity. The potential biostimulating effects have so far been underreported. The assessment of the stimulating effect of botanical biomaterials is essential in the cultivation of economically-important crops. An attempt was undertaken in this study to develop a new biostimulating material in the form of granules, based on an extract from the roots of *Arctium lappa* L. The scope of the research included the characterization of the new material and the identification of its biostimulating potential. The designed and produced biogranulate is rich in bioactive compounds, including polyphenolic compounds, carbohydrates, and micro- and macro-elements. The analysis of the physicochemical properties of the biomaterial has shown that it had the features of intelligent biopreparations, i.e., slow-release preparations, at the pH appropriate for legume plants. Thus, knowledge about the design of new biomaterials is a milestone in the practical development of new perspectives for enhancing sustainability in agriculture.

## 1. Introduction

Synthetic chemical fertilizers are commonly used in agricultural practice as inexpensive agents, ensuring the immediate availability of nutrients to crops. However, a growing awareness of the population has been observed over the years regarding the adverse effects of the excess and irrational application of chemical fertilizers, leading to increased emissions of greenhouse gases and to soil degradation [1], and the eutrophication of water masses [2]. The first attempts of using organic fertilizers (manure or post-harvest residues) proved that, although in some way, they can make agriculture independent of chemical fertilizers, they still fall short in sufficiently meeting plant demands for nutrients and minerals [3].

The use of biostimulants, representing a group of plant growth and development promoters not defined as fertilizers, seems to offer a prospective approach to aid sustainable agriculture. Biostimulating agents include, i.a., extracts from seaweeds, protein hydrolysates, and plant extracts. Investigations concerning plant extracts and focusing mainly on the extraction of bioactive compounds intended for pharmaceutical, food, or medical industries have recently spurred great interest. However, the key area of scientists’ explorations has been the development of environmentally-friendly solutions (biostimulants) based on natural products, like secondary plant metabolites [4].

Cutting-edge methods harnessed to seek novel solutions for agriculture draw from the principles of allelopathy, i.e., biochemical interactions between various plant classes expressed in the production of chemical compounds [5]. Until now, because of the wide availability of synthetic chemical compounds or long-term exploitation of marine algae, scientists who assessed the effects of biostimulants on crops ignored the stimulating effects of allelopathic plant extracts. Nevertheless, some reports have demonstrated plant stimulation by other plants, as corroborated in previous research conducted by our research group. Extracts from allelopathic plants have been shown to improve the germination and development of seedlings of many crops [6,7,8,9,10]. In addition, when used via foliar or soil application, plant extracts have also affected the seeds yield of soybeans and biometric traits (plant height, location height of the first pod, 1000 seeds weight, and number of pods) [10,11]. Other literature confirms the feasibility of using plant extracts in crop cultivation under conditions of salt stress, proving they represent effective natural biostimulants [12,13,14,15,16,17]. 

However, there is still paucity of research into the use of plant extracts as natural growth and development stimulants in crop cultivation [18]. Therefore, the development of novel biomaterials, based on plant extracts, is expected to boost yields without adversely affecting environmental diversity, and also to reduce the use of chemical fertilizers [19]. It needs to be emphasized that the biostimulating effect of such products is chiefly due to the presence of various classes of molecules in allelopathic or medicinal plants. Secondary plant metabolites, representing the major group of bioactive compounds, are of key importance. They not only elicit a biostimulating effect on crops, but also protect crops from phytopathogens [4].

Multiple factors ought to be considered when designing a novel biostimulating material for agriculture, including the form and method of its application, in order to ensure its efficacy. Biostimulants can be used as seed dressing agents, soil additives, or spraying solutions. They can be produced in the form of fluids, concentrates, granulates, or powders [20,21]. However, they should share a common feature, namely be derived from raw materials rich in bioactive compounds. In many cases, the mechanism of their action has remained unresolved, probably due to their complicated chemical composition, or to the potential additive or synergistic effects of their components [20,22]. 

The application of biostimulants can induce the primary or secondary metabolism of crops, ultimately leading to an increased effectiveness of nutrient utilization, and to the modulation of the nutritive value and chemical composition of finished products [23,24,25,26]. Hence, the positive biostimulating outcomes of these products are due to the activation of a complex signaling mechanism of crops, whose role is to induce an appropriate response to the received stress stimuli by, e.g., stimulating the antioxidative system or photosynthetic apparatus [27]. Therefore, when designing novel biostimulating materials, caution should be exercised regarding the multiple allelochemical-induced physiological activities of crops responsible for the growth responses of receiver plants [28].

The success in the practical application of modern botanical biostimulants is largely driven by their bioactive compounds and their biocompatible form, which not only ensure plant growth and development and stimulate yield, but additionally enable their convenient application. Only this approach may incline farmers to replace chemical fertilizers and stimulants with natural alternative counterparts. Problems with biostimulant application can be overcome by implementing an appropriate formulation technology. Granulated biostimulating formulations seem to offer an intriguing system of delivering bioactive ingredients to crops because they combine defined effects with limited environmental impact. Hence, importantly for farmers, it is possible to deliver the biostimulant in the right amount, at the right place, and at the right time [29]. The second factor determining the introduction of new products to agricultural practice is the effectiveness of their operation, mainly related to the content of bioactive compounds. 

Among plants rich in secondary metabolites with an allelopathic activity worthy of notice is the greater burdock (*Arctium lappa* L.), belonging to the Asteraceae family. Its choice for this research was driven by many years of investigations in other fields of science (food technology, medicine, and cosmetology), demonstrating multiple biological activities of its root extracts, like antipyretic, antimicrobial, antioxidative, anti-inflammatory, and anticarcinogenic effects [30,31,32,33,34]. Greater burdock has been used as the main component of many pharmaceutical preparations [35]. Literature works report multiple health benefits ascribed to various classes of its secondary bioactive metabolites [31]. Studies on the use of greater burdock extracts in improving the germination of cereal grains, as well as vegetable and oilseed seeds, have also shown its activity against phytopathogens. It was found that the degree of infestation of the germinating plants was significantly reduced compared with the control samples [6,7]. However, the potential biostimulating effects of its extracts have so far been underreported, which is all the more striking considering its rich composition.

Given the above statements, an attempt was undertaken in this study to develop a new biostimulating material in the form of granules, based on an extract from the roots of *Arctium lappa* L. A research hypothesis was thus advanced that the botanical biostimulant rich in bioactive compounds, including particularly polyphenolic compounds, would offer a novel agronomic strategy to improve the effectiveness of crops. The scope of the research included the characterization of the new material and the identification of its biostimulating potential. This integration of analytical methods is expected to allow for the practical development of future perspectives for enhancing sustainability in agriculture.

## 2. Materials and Methods

### 2.1. Botanical Extract Production

Extracts were prepared from dried, ground roots of *Arctium lappa* L., originating from biofarming (Runo Poland, PL-EKO 04 EU Organic Farming). Infusions from burdock were prepared using the hot extraction method, i.e., 10 g of ground roots of *Arctium lappa* L. were added to 250 mL of distilled water. The solution was boiled in a water bath for 30 min. Afterwards, the extract was centrifuged at 4250 rpm for 5 min and filtered through a blotted filter paper of Whatman no. 1, for sterile polystyrene sample containers with a screw cap (polyethylene/polypropylene) (Witko, Łódź, Poland) [36].

### 2.2. Preparation of Granulated Biomaterial

**Figure 1:**
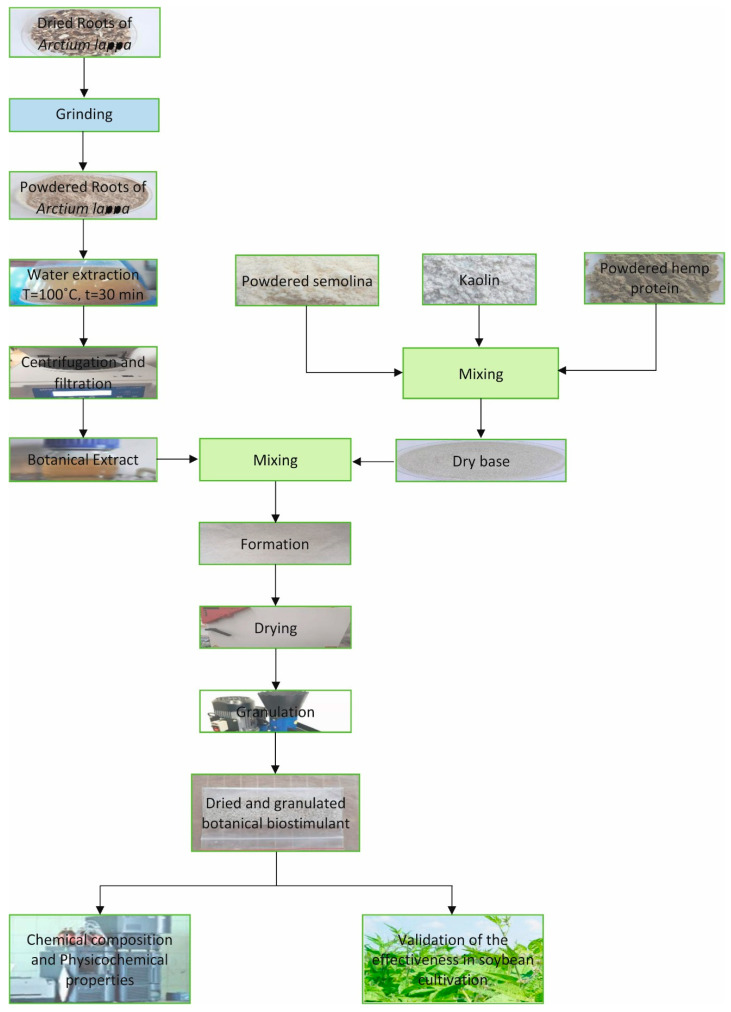
Proposed flowchart of botanical biomaterial production and validation.

Granulate was prepared according to the modified procedure described by Zahran et al. [29]. First, 128 g of organic flour from durum wheat (Semolina, Bioplanet, Leszno, Poland, PL-EKO 07 EU Organic Farming), 24 g of kaolinite (white clay, Cosmo, Poland), and 8 g of hemp protein powder (Bioplanet, Leszno, Poland, PL-EKO 07 EU Organic Farming) were mixed in a beaker. Next, 92 mL of a botanical extract from *Arctium lappa* L. roots were added to the beaker, and all of the ingredients were placed in an automatic pasta sheet machine with a dough mixer A160 K DV (Capitani snc, Lomazzo, Italy). The resulting 1-mm thick sheets were pre-dried in a laboratory drier with forced convection (Binder ED53, Tuttlingen, Germany) to a water content of 20%. Next, the material was placed in a Rotex-100 granulation machine (LabEcoTech, Konstancin-Jeziorna, Poland) (Figure 1).

### 2.3. Physicochemical Properties of Granulated Botanical Biostimulant

The pH values were measured using a VOLTCRAFT KBM-110 m (Conrad Electronic SE, Hirschau, Germany) with a pH electrode [37]. The rate of granule dissolution was determined as the ratio of the weight of granules dissolved in water flowing through a column to the dissolution time (s). The rate of biomaterial dissolution was determined with the conductometric method by measuring the electrical conductivity of the resulting solution. The dissolution process was analyzed using a CC502 conductometer (Elmetron, Zabrze, Poland). The rate of distilled water flow through the system (0.93 mL/s) was determined before the measurements of the dissolution rate. The analysis of the granulated biomaterial dissolution rate consisted of placing a 2-g sample on a teflon sieve mounted on a column through which distilled water was flowing at a constant rate. The column was mounted in such a way that the water level in the system was at the same height as the outlet opening in the measuring probe. This ensured the same initial conditions for each of the measurements. The measurement of the specific conductivity of the filtrate began when the test sample was placed on the sieve, and proceeded via automatic recording of the results on the computer the conductometer was connected to. The flow of distilled water through the column resulted in fertilizer dissolution. The resulting solution washed the flow-through conductometric probe coupled to the conductometer, allowing for the registration of changes in specific conductivity over time. The filtrate was collected to a beaker placed under the probe. The process was carried out for one hour and the conductivity coefficient was read every 60 s. Afterward, the beaker with the filtrate was removed. The remaining undissolved fertilizer was transferred to the second beaker, and the sieve was rinsed with distilled water. The obtained filtrate and the residual undissolved biomaterial were weighed on an analytical balance, then placed in a drier, and dried at 110 °C for 24 h to a constant weight. The above procedures allowed for computing the amount of the dissolved substance and the weight loss of the granules [38].

### 2.4. Chemical Composition of Granulated Botanical Biostimulant

All samples of the granulated botanical biostimulant were prepared under a high pressure microwave digestion system, in 65% extra pure nitric acid. First, 0.5 g of samples were put in digestion vessels and filled up with 8 mL of nitric acid as a reagent. The same procedure was used with a blank sample (acid and water clean control procedure). An Ethos One microwave digestion system (Milestone, Italy) was used during the digestion procedure. The samples were digested at an algorithm of temperature, increasing as specified for biological samples, never exceeding 200 °C. After all of the samples were filled up to 50 mL with deionization water (<0.07 µS cm^−1^), the detection threshold was better than 0.01 mg kg^−1^ for each element (spectrometer detection capacity is over 1µg L^−1^). The measurements were performed with ICP-OES (Inductively Coupled Plasma Optical Emission Spectrometers, Thermo iCAP Dual 6500, Antigo, WI, USA). Each measurement could be made in two planes of the plasma flame (Radial and Axial). Calibration curves were created in two concentration variants by certified Merck models. The calibration curve was built on the basis of three concentration points. For the measurements, especially optical correctness, internal standards in the form of Y and Yb were used (Y = 2 mg L^−1^ and Yb = 5 mg L^−1^ [39].

Sugars in the granulated biomaterial were examined based on the procedure of Crha and Pazourek [40], using the HPLC system. For the analysis a Sugar-Pak I, 10 μm, a 7.8 mm × 300 mm analytical column was used (Rezex RCM-Monosaccharide Ca^2+^ for use with monosaccharides and sugar alcohols, including sorbitol and mannitol from sweeteners and corn). Separation was performed in 25 min, at a flow rate of 0.5 mL/min and a column temperature of 80 °C. The extracts were eluted using 0.1 mM calcium disodium EDTA. The compounds were quantified using a refractive index detector RID-10A, as an element of the HPLC system (Shimadzu, Kyoto, Japan).

The total fat content was determined according to the acid hydrolysis method (AOAC, 2000, Official Method 922.86) [41]. The total protein content was determined by the Kjeldahl method (AOAC, 2000, Official Method 992.23, 979.09) [41]. The total protein and fat content was expressed in % of dry matter (DM) in granules.

### 2.5. Microbiological Analyses of Non-Microbial Granulated Biostimulant

*Salmonella* spp. was estimated in a simplified version of EN ISO 6579-1:2017. Initial growth was carried out in buffered peptone water for 16 h at 37 °C. Selection was made in tetrathionate broth according to Müller Kauffmann (Merck, Darmstadt, Germany) after 24 h at 43 °C. Subsequent demonstration was done on brilliant green agar after 24 h at 37 °C through conformational agglutination and biochemical reactions [42].

A horizontal method for the enumeration of beta-glucuronidase-positive *Escherichia coli* was used according to the ISO 16649-2:2001 on TBX (Tryptone Bile X-Glucuronide Agar) (BioRad, Hercules, CA, USA) after incubation at 44 °C for 24 h [43].

### 2.6. Statistical Analysis

The statistical analysis of the results was carried out in Statistica 13.3 software (TIBCO Software Inc., Palo Alto, CA, USA). In the case of assessing the chemical composition of the biomaterial, the value of the standard deviation was given (±SD).

## 3. Results and Discussion

The evaluation of the physicochemical properties of the produced biomaterial demonstrated its active acidity was 6.05. This value is consistent with values reported for standard agricultural products classified as agrochemicals. Oancea et al. [37] made similar observations when producing a biostimulant with *Trichoderma* strains. Their investigation showed a pH of approximately 6 to be optimal to ensure desired effects of novel biomaterials in real field conditions. Such a pH value is also essential to legumes, like soybean, which strongly respond to acidification, with optimal pH values for their growth and development ranging from 6.0 to 7.5 [44].

The analysis of biostimulating granulate properties demonstrated that its dissolution rate was 0.021 g/min, while its solubility reached 39.7%. This indicates that the developed biomaterial has some features of encapsulated granular chemical fertilizers. Hence, its application in agricultural systems should not differ from that of the agrochemicals used so far, even though the determined values of its parameters indicate it shares some features of a slow-release fertilizer of organic origin. Therefore, it can be expected that its application in real field conditions will elicit a stimulating effect, spread over time, which in turn will provide a longer availability of biostimulating compounds to developing plants [45].

**Figure 2:**
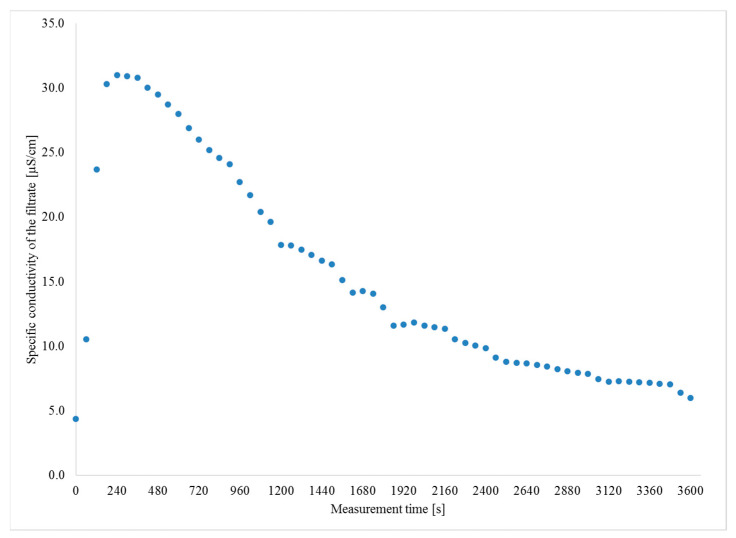
Specific conductivity of the filtrate.

Evaluation of the specific conductivity of the filtrate, performed during the analysis of the biomaterial solubility, demonstrated its maximum values at the initial stage of the process (between 180 and 240 s) (Figure 2). Afterwards, the specific conductivity of the filtrate, formed after biogranulate dissolution, decreased slowly to finally stabilize between 3360 and 3600 s of the measurement. These observations may point to the first success in producing novel biomaterials for agricultural purposes, because the botanical biogranulate produced in this study shows some features of intelligent fertilizers, i.e., slow-release fertilizers. In addition, this corroborates the findings reported by Bortolin et al. [46], who proved that the use of kaolinite led to some improvement in the properties of fertilizing materials. Hence, these first results indicate an excellent potential of kaolinite in producing innovative controlled-release or slow-release fertilizers [47].

Fourteen polyphenolic compounds with different retention times were quantified in the botanical biogranulate from *Arctium lappa* roots. Among these identified polyphenols in the biogranulate, based on the infusion of burdock roots, the following dominated: 1,4-dicaffeoyl-3-maloylquinic acid (61.09 ± 0.32 μg/g) ascribed to its deprotonated pseudomolecular ion [M-H] at *m*/*z* 631, and its fragments with *m*/*z* 469, 353, and 191 at the absorption maxima between 298 and 325 nm, and 3,4-dicaffeoyl-5-succinoylquinic acid (54.47 ± 3.71 μg/g), ascribed to its deprotonated pseudomolecular ion [M-H] at *m*/*z* 615, and its fragments with *m*/*z* 515, 353, and 191 at the absorption maxima between 298 and 325 nm. The botanical biogranulate was also rich in chlorogenic acid (25.10 μg/g of chlorogenic acid) and 1,3-dicaffeoyl-4,5-dimaloylquinic acid (20.02 μg/g) (Table 1).

The botanical biogranulate also contained caffeoylquinic acid derivatives (in concentrations approximating 12 μg/g) that were identified based on deprotonated pseudomolecular ions [M-H] with *m*/*z* 631, 731, and 715 (1,5-dicaffeoyl-4-maloylquinic acid, 1,5-dicaffeoyl-3-succinoyl-4-maloylquinic acid, and 1,5-dicaffeoyl-3,4-disuccinoylquinic acid, respectively). Furthermore, the botanical biogranulate had high concentrations of phenolic compounds, with their total content reaching 241.30 μg/g. Such a content of bioactive compounds is beneficial, considering the expected biostimulating effect of the biogranulate on plant growth and development, because phenolic compounds affect seed germination, cell division, biomass accumulation, or plant metabolism improvement [48,49,50]. Hence, the polyphenolic acid composition determined in the present study allows for speculating that the biogranulate produced will influence the key metabolic and physiological processes in plants at early stages of their development [51]. When applied in appropriate doses, polyphenolic compounds not only serve as growth promoters, but also play an essential role in improving plant tolerance and capability to adapt to stress conditions [52]. Therefore, preparations containing bioactive compounds can be classified among natural plant biostimulants. In addition, polyphenolic acids are involved in nutrient supply, which is especially important while designing novel multi-component biostimulants.

Table 2 presents the multi-element composition of the botanical biogranulate, based on the extract from greater burdock roots. The study results indicate that the botanical biogranulate is rich in both macro- and micro-elements. It has high contents of potassium, calcium, and magnesium, and no toxic elements. Particularly noteworthy is the high content of potassium, which—together with magnesium—is involved in the photosynthesis process leading to the production of carbohydrates [44]. The analysis of the composition of macronutrients coupled with the identification of polyphenolic acids allows for putting forward a hypothesis that the biogranulate produced will have a biostimulating effect on plants, because polyphenols increase the rate of nutrient uptake by chelating metal ions, the number of active absorption sites, and soil porosity, while simultaneously accelerating the mobilization of such elements as calcium, magnesium, potassium, zinc, and iron [50,53].

The determination of the contents of micro-elements yielded results, as the biogranulate turned out to be a rich source. It contained, iron, copper, manganese, zinc, and cobalt, with a zinc concentration reaching 27.29 mg/g.

Therefore, it can be concluded that the botanical biogranulate, based on active compounds from the roots of greater burdock, contains a pool of elements that play role in ensuring the proper growth and development of plants due to the influence of micro- and macro-elements on various life processes. The elements can stimulate the mechanisms of plant resistance to biotic and abiotic stress, or support the uptake of other nutrients [54].

The botanical biogranulate also contained protein and fat at levels of 6.581% and 1.022%, respectively (Table 3). Such a composition seems beneficial for plant biostimulation, because the amino acids of biostimulants are mainly precursors of polyamides responsible for cell division and precursors of lignin biosynthesis [55]. The protein contained in the biogranulate may influence the biosynthesis of many different compounds necessary for plant development (including nucleotides, chlorophyll, hormones, and secondary metabolites). In turn, the delivery of lipids, including fatty acids, with the preparation is inherently associated with making membranes fluid and stimulating plant mechanisms of adaptation to stress conditions.

The assessment of the chemical composition of the botanical biogranulate based on active compounds from the roots of the greater burdock showed the presence of carbohydrates, i.e., sucrose, glucose, and fructose (Table 3). The biomaterial also had a high concentration of sucrose. A slightly lower concentration was determined for glucose. Apart from polyphenolic acids and micro- and macro-elements, the additional carbohydrate content in the botanical biogranulate will determine its biological activity in plant biostimulation. According to Kumar et al. [56], this activity may be related to the latest research results indicating carbohydrates’ role in inducing the so-called “sweet immunity”. This theory assumes that plant resistance can be controlled and regulated by sugars and their derivatives, thus contributing to the optimal functioning of the defense system [56]. Therefore, a botanical biogranulate containing a carbohydrate pool will most likely affect the primary antioxidant system of plants, the activity of which allows plants to combat stress by maintaining the cellular balance between reduction and oxidation reactions [28].

The assessed composition of the botanical biogranulate proves that it is possible to produce a complex system of bioactive compounds, with the content of polyphenolic acids and carbohydrates being one of its strongest advantages. According to Rolland et al. [57], these compounds exhibit a high biological activity, by, e.g., regulating an increase in the volume of plant cells, their division, and development. 

Research has shown that pathogens in the non-microbial biostimulant do not exceed the permissible levels (Table 4) specified in regulation EU 2019/1009 of the European parliament and of the council [58]. Thus, the tested granulated non-microbial biostimulant can be considered as a safe agronomic solution and can be subjected to further investigations in the future.

## 4. Conclusions

Summarizing the conducted research, it can be stated that the designed and produced botanical biogranulate, based on the extract of *Arctium lappa* L. roots, is rich in bioactive compounds, including polyphenolic compounds, carbohydrates, and micro- and macro-elements. The analysis of the physicochemical properties of the novel organic biomaterial has shown that it had the features of intelligent biopreparations, i.e., slow-release preparations, at a pH appropriate for legume plants. Thus, the knowledge about the design of new biomaterials is a milestone in the current and future practical development of new perspectives for enhancing sustainability in agriculture. However, further research in this area is needed, especially concerning the response of plants at many levels, including biochemical or molecular ones, which, in turn, will enable progress in the design of new biomaterials for agricultural purposes.

## Figures and Tables

**Table 1:** Individual and total phenolic compounds identified by UPLC-PDA-MS/MS in the botanical biogranulate (average ± SD).

No.	Compound	Rt	λmax	[M-H] *m*/*z*		Polyphenols Content
		min	nm	MS	MS/MS	μg/g
1	Chlorogenic acid	2.91	298 sh. 325	353	191. 179	25.10 ± 1.57
2	Cryptochlorogenic acid	3.04	298 sh. 325	353	191. 134	3.01 ± 0.23
3	3-*O*-feruloyl-quinic acid	3.65	298 sh. 325	367	193. 173	0.61 ± 0.01
4	1.5-dicaffeoyl-3-maloylquinic acid	4.72	298 sh. 325	631	469. 353. 191	7.23 ± 0.18
5	1.4-dicaffeoyl-3-maloylquinic acid	5.00	298 sh. 325	631	469. 353. 191	61.09 ± 0.32
6	1.5-dicaffeoyl-quinic acid	5.12	298 sh. 325	515	353. 191	13.79 ± 0.57
7	1.3-dicaffeoyl-4.5-dimaloylquinic acid	5.17	298 sh. 325	747	631. 469. 353	20.02 ± 0.49
8	1.5-dicaffeoyl-4-maloylquinic acid	5.20	298 sh. 325	631	469. 353. 191	12.63 ± 0.38
9	3.4-dicaffeoyl-5-succinoylquinic acid	5.39	298 sh. 325	615	515. 353. 191	54.47 ± 3.71
10	1.5-dicaffeoyl-3-succinoyl-4-maloylquinic acid	5.53	298 sh. 325	731	631. 469. 353	12.63 ± 0.38
11	3.4-dicaffeoyl-3-succinoyl-4-maloylquinic acid	5.63	298 sh. 325	731	631. 469. 353	9.30 ± 0.08
12	3.4-dicaffeoyl-5-succinoylquinic acid	5.80	298 sh. 325	615	515. 353. 191	6.64 ± 0.02
13	1.5-dicaffeoyl-3.4-disuccinoylquinic acid	6.09	298 sh. 325	715	553. 453. 353	12.12 ± 0.13
14	Tri-caffeoyl-succinoylquinic acid	6.58	298 sh. 325	777	497. 353. 191	2.77 ± 0.29
Total (μg/g)						241.30 ± 3.39

**Table 2:** Multielemental composition of the botanical biogranulate (average ± SD).

Element	Content
Macroelements, mg/g	
Calcium, Ca	53.18 ± 0.25
Potassium, K	869.60 ± 24.99
Magnesium, Mg	40.67 ± 0.61
Sodium, Na	26.46 ± 0.19
Phosphorus, P	39.32 ± 0.67
Sulfur, S	28.74 ± 0.63
Microelements mg/g	
Aluminum, Al	<LLD
Chrome, Cr	<LLD
Cooper, Cu	0.124 ± 0.011
Iron, Fe	2576.92 ± 90.37
Manganese, Mn	0.151 ± 0.007
Molybdenum, Mo	<LLD
Nickel, Ni	<LLD
Strontium, Sr	0.583 ± 0.061
Zinc, Zn	27.29 ± 0.59
Cobalt, Co	1.057 ± 0.071
Toxic metals mg/g	
Arsenic, As	<LLD
Cadmium, Cd	<LLD
Lead, Pb	<LLD
Active acidity	
pH	6.05 ± 0.02

<LLD—below limit of detection.

**Table 3:** Chemical composition of the botanical biogranulate (average ± SD).

Total Protein	Total Fat	Carbohydrates			
		Saccharose	Glucose	Fructose	Sorbitol
%	%	mg/g			
6.581 ± 0.122	1.022 ± 0.021	3.097 ± 0.025	2.733 ± 0.069	0.834 ± 0.011	<LLD

<LLD—below limit of detection.

**Table 4:** Microbiological analyses of non-microbial granulated plant biostimulant.

Tested Microorganisms	Maximum Value of the Number of Bacteria Expressed in CFU Acc. [58]	Non-Microbial Granulated Biostimulant Expressed in CFU
*Salmonella* spp.	Absence in 25 g or 25 mL	0
*Escherichia coli*	1 000 in 1 g or 1 mL	0

## Data Availability

Data is contained within the article.
